# Redesigning community care for safer staff and patient experiences: quality improvement project to improve safety and reduce incidents of violence and aggression in a community mental health team

**DOI:** 10.1192/bjo.2021.580

**Published:** 2021-06-18

**Authors:** Sarah Saxena, Alberto Gutierrez Vozmediano, Katrina Walsh, Sarah Moodie, Rumbi Mapfumo

**Affiliations:** South West London and St George's Mental Health NHS Trust

## Abstract

**Aims:**

Violent or aggressive incidents can be relatively common in community settings, and perhaps more difficult to manage than at inpatient wards due to the relative isolation and peripatetic delivery model, which can put staff at higher risk during incidents. Carshalton and Wallington Recovery Support team was identified as an outlier in the Trust and was invited to partake in a Safety Collaborative across South London Partnership.

Stakeholders agreed on the aim of reducing incidents by 20% over 1 year by the end of 2020.

**Method:**

Data about incidents were analysed and staff surveys conducted to evaluate violent events. Patient discharge was highlighted as a particular time of increased aggression. Involvement of patients and carers through patient focus groups and co-production was essential to elicit areas of improvement. These included staff confidence and awareness of existing guidelines. Additional secondary drivers were communication with patients, care pathway development, discharge process and multidisciplinary approach, which each had associated change ideas.

The team identified change ideas that have been tested over one year using the Quality Improvement methodology of small-scale testing and PDSA. Example ideas tested include multidisciplinary Risk meetings, Safety huddle tool, Staff Safety training, co-produced Welcome and Discharge Packs with informed care pathways.

**Result:**

There has been a 30% reduction in incidents by December 2020 across a total of 280 patients. Surveys have shown an increase in staff confidence and safety protocol awareness from 40% to 70% by October 2020. 100% of patients in focus groups found the Welcome and Discharge Packs helpful.

**Conclusion:**

A structured improvement approach focused on staff safety and minimisation of known and potential contributing factors can lead to a reduction in incidents. Safety huddles and risk meetings allow a formal multidisciplinary approach to management of violence and aggression. Staff feel more reassured about safety policies in the trust, with better communication between senior management and colleagues to highlight risk and provide support. A culture of open discussion and transparency was implemented through provision of Welcome Packs including Care and Discharge Pathways details at point of entry to the service. Support was provided to patients with Discharge Packs including information about community services. This enabled a meaningful support model at the end of their recovery journey and an improved discharge process.

The team is now working with additional teams and administrative and clerical staff to improve safety. We hope to replicate this approach in our Trust.

